# The doctor-patient relationship: toward a conceptual re-examination

**Published:** 2016-08-28

**Authors:** Hamidreza Namazi, Kiarash Aramesh, Bagher Larijani

**Affiliations:** 1 PhD Candidate in Medical Ethics, Medical Ethics and History of Medicine Research Center, Tehran University of Medical Sciences, Tehran, Iran;; 2 Associate Professor, Medical Ethics and History of Medicine Research Center, Tehran University of Medical Sciences, Tehran, Iran;; 3 Professor, Medical Ethics and History of Medicine Research Center, and Endocrinology and Metabolism Research Institute, Tehran University of Medical Sciences, Tehran, Iran.

**Keywords:** *Doctor-patient relationship*, *Philosophy*, *Psychology*, *Sociology*, *Orbital parameters*

## Abstract

The nature of the doctor-patient relationship as a keystone of care necessitates philosophical, psychological and sociological considerations. The present study investigates concepts related to these three critical views considered especially important. From the philosophical viewpoint, the three concepts of "the demands of ethics “,” ethical phenomenology and "the philosophy of the relationship" are of particular importance. From a psychological point of view, the five concepts of "communication behavior patterns" (including submissiveness, dominance, aggression, and assertiveness), "psychic distance", "emotional quotient", "conflict between pain relief and truth-telling", and "body language" have received specific emphasis. Lastly, from the sociological perspective, the three notions of "instrumental action", "communicative action", and "reaching agreement in the light of communicative action" are the most significant concepts to reconsider in the doctor-patient relationship. It should be added, however, that from the sociological point of view, the doctor-patient relationship goes beyond a two-person interaction, as the moral principles of doctors and patients depend on medical and patient ethics respectively. The theoretical foundations of the doctor-patient relationship will finally help establish the different dimensions of medical interactions. This can contribute to the development of principles and multidisciplinary bases for establishing practical ethical codes and will eventually result in a more effective doctor-patient relationship.

## Introduction

The doctor-patient relationship plays an essential role in ordering the health care system and medical ethics, and since it is a form of communication, it necessitates ethical, philosophical, psychological, and sociological considerations. The present paper aims to evaluate the essence of the doctor-patient relationship in order to re-examine its conceptual framework. In the first part, the philosophical, psychological and sociological significance of this relationship is explored, and in the final section, the theoretical implications will be discussed. It seems that despite the imbalance in the relationship between doctors and patients resulting from the greater significance of the physicians’ ethics, organization of this relationship is not possible without enhancing patient ethics.

Simultaneous consideration of sociological, psychological and philosophical dimensions of the doctor-patient relationship can contribute to developing theoretical foundations and multidisciplinary bases for establishing practical ethical codes. The result will eventually be a more effective interaction between the two.

**A) The Philosophical Essence of the Doctor-Patient Relationship**

In investigating the philosophical essence of the doctor-patient relationship, three points should be taken into consideration. First, ethical demands in doctor-patient interactions must have distinct definitions and terms; second, the phenomenological ethical debates on this issue need to be explored; and third, modern topics in the philosophy of the relationship should be considered, and relationships with the others should be analyzed from different perspectives.

***Ethical Demands***

Various organizations and professions differ in their attitudes towards ethical demands, recommendations, norms, values and judgments. The three components of inclusion, priority and severity are presented below as the criteria for judgment in ethical issues.

**1. Inclusion: **The main questions to answer in regard to this component are: "What are the ethical limits?” and “Should all of our actions be judged ethically or only some of them are included in the scope of ethical judgment?" In other words, is it enough to avoid doing the wrong thing, or is doing right among our moral duties too? It seems that the doctors’ moral duties include doing the right thing as well. This important matter is embedded within the principles of beneficence and non-maleficence.

**2. Priority: **The component of priority relies on the answers to the following questions: "If what morality is demanding is in conflict with our personal interests (for example it concerns our self, family, friends and so on), which side should we take? Should we always take the ethical side and forget about our personal interests? Or personal interests could have priority over moral obligations?" Nigel and Stalker explain that autonomy and our personal integrity have priority over what morality is demanding from us, or as Kagan (1) and Singer (2) say, demandingness of morality can even affect autonomy and our personal integrity. It seems that on the one hand the altruism of a practitioner as a professional should be based on the priority of patients’ interests, and on the other hand it should safeguard the practitioner’s own autonomy.

**3. Severity: **The main questions here are: Can ethics press extreme and costly demands from us? Or are the obligations of morality lighter and easier in the way that most people could overcome?" Apparently if ethics are founded on costly demands, we will be more likely to fail to fulfill our ethical duties.

Based on the above-mentioned considerations and classifications, three macro-positions emerge in the ethical relationship, including: maximal ethics, ordinary ethics, and minimal ethics.

*Maximal Ethics*: Maximal ethics include all the three components discussed above. In this type of ethics, ethical inclusion does not have any limits and covers all human actions. Extremist moralities consider ethical inclusion to be an absolute matter that covers all life styles and signify that no human action should be outside of this infinite circle.

*Ordinary Ethics:* This is the sort of ethics that most people believe in, and because of its affinity to the contemporary human life, it is also referred to as “common ethics”. Here what ethics demands from us are boundaries. In other words, moderate ethics often state that after performing our obligations and moral duties, in a relatively wild range of personal interests we can start selecting. Thus, our actions are not always subject to moral judgment.

*Minimal Ethics* **: **This type of ethics is contradicted with maximal ethics. According to minimalists, the only forbidden action is intentional harassment. Followers of minimal ethics believe in a wide range of choices and selection areas; they recognize only a limited range of constraints and are in favor of acting upon personal interests (3).

It is a growing concern in medical ethics that the doctor-patient relationship is not approached in a sufficiently broad way and that this overly narrow medical perspective leaves doctors, nurses and other health care professionals badly equipped to deal with ethical dilemmas (4). Phenomenology could broaden this perspective and serve as a strong basis to understand moral sensitivity. Two notions in phenomenology have a central role in understanding the concept of the doctor-patient relationship: intentionality and first-person point of view.

***Intentionality and first-person point of view***

One of the basic concepts of phenomenology is attainment of phenomenal intentionality, which occurs when a person recognizes earlier assumptions and adopts a perspective (5). Some thinkers like Franz Brentano believe that intentionality and the phenomenological approach can be applied to the first-person point of view (6). For instance the first sighting of a beautiful landscape elevates us in a way that may not happen in later encounters. The reason is that later encounters are accompanied by presuppositions of the observer, who will be more used to the landscape. It seems that the phenomenological approach can be applied to the doctor-patient relationship. Doctors must reexamine and restrict assumptions toward patients, and at the same time value intentionality in order not to fall into habits.

***Moral Sensitivity***

Moral sensitivity may be enhanced in two ways. First, through reinforcing the phenomenological approach by renewing the first sight experience, that is, in each re-identification (of the patient for instance), priorities should be observed. Second, since any situation could come to a fork and ethical conflicts may rise, the adverse impacts should be considered and every situation must be regarded from an ethical perspective. Although at commencement moral sensitivity appears to overlap with maximal ethics, it is of particular importance especially in heterogeneous communications such as the doctor-patient relationship. It may be added that enhancing moral sensitivity even seems to be the target of the phenomenology of ethics in the doctor-patient relationship (7).

***Communication with Others***

The term “communication” can be defined through the philosophical approaches of great thinkers such as Levinas, Marcel and Buber who set their philosophical arguments in the relationship between “me” and “the other”. Levinas insists on the maximum responsibility of any other; Marcel assists on turning the me-that relationship to the me-you relationship and replacing absence with presence; Buber finds God in "Thou”. 

**B) The Psychological Essence of The Doctor-Patient Relationship**

In terms of psychology, the doctor-patient relationship is imbalanced as the doctor has superiority over the patient. Such imbalanced relationships may give rise to various patterns of communication behavior. Psychologists (8) have distinguished the following four communication behavior patterns based on components such as honesty, perspicuity, respect and inhibition:

*1) Submissiveness*

Submissive persons are shy, and although they speak honestly, they are usually taciturn and cannot express themselves perspicuously. They are also afraid of being judged or offending others, so they are incapable of making eye contact while speaking. Their voices are weak and unsteady, and they speak hesitantly. Submissive people avoid conflict rather than try to resolve it. They speak indirectly and in general terms because they cannot express themselves openly and may quickly feel depressed and vulnerable. People with this behavioral pattern admittedly let others abuse them and treat them disrespectfully. These patterns work both for doctors and patients. Patients who evade their responsibilities and encourage physicians to patriarchy in the process of therapy, or doctors who are not able to say “no" to patients easily consent to inappropriate and ineffective treatments.

*2) Dominance*

Domineering people feel insecure and believe that they do not possess good qualities. Accordingly, they try to deceive others and take advantage. Domineering persons do not have the perspicuity and honesty necessary for earning their wishes. They express themselves in general terms and sometimes their voices shake. These people use others to achieve their goals and make light of this inhibition and deception, so they take away another person’s autonomy and freedom. This behavioral pattern is often seen in doctors and sometimes among patients as well. Doctors who prefer patient satisfaction to authority thus create a false autonomy for the patients and will eventually be dominated by them, and patients with this behavioral pattern impair the healing process by inhibition and deception.

*3) Aggressiveness*

The target of aggressive and domineering people is very similar and that is exploitation and domination of others. Their difference is that a domineering person achieves this aim by secrecy and cheating, while an aggressive person follows it frankly and openly. Unlike the domineering type, aggressive people are honest and straightforward; they are horrible listeners, always accuse others, get angry soon, get confused by criticism, and are usually grim in appearance. They have loud voices and look hostile, and in conflicts, they tend to destroy their opponents. This pattern is seen among both physicians and patients. Impatient physicians that do not listen, shout all the time and sometimes make irreparable mistakes during the healing process, or patients with lower anger thresholds who create tension in medical environments belong in the category of aggressive people. 

*4) Assertiveness*

Assertiveness is the most creative behavioral pattern of communication. Assertive people respect themselves and others, and observe the authority of all sides. They are both honest and frank, and do not accuse themselves or others. Their approach to matters is problem-oriented, that is, when dealing with a problem, instead of accusing themselves and others, they think of a solution. They listen effectively and speak appropriately and understandably. During conflict they emphasize conversation. Their arguments are clear, specified, objective, fair and respectful, and eventually they are the most successful communicators. Issues such as breaking bad news, wasted treatments and medical mistakes are easy and solvable with this type of behavioral pattern. While submissiveness, dominance and aggression lead to lose-lose situations in long term, assertiveness, is a helpful behavioral pattern and finally results in win-win solutions (9).

Based on the above-mentioned notions, the following practical hints should be outlined: 

**1. **Psychic Distance**: **An important topic in aesthetics and artistic criticism that is also related to ethics is psychic distance. In aesthetics, this refers to the distance that should exist between a work of art and the viewer, so that aesthetic entente is created and art is not confused with reality. Omitting the psychic distance and forming deep sympathy and psychological identification with the work of art obstructs artistic judgment and aesthetic approach. In medical ethics, the concept seems to be important while encountering patients. Reduction of psychic distance and excessive sympathy with patients prevent an effective doctor-patient relationship as a fundamental element of treatment. 

**2. **Body Language: Nonverbal communication skills are referred to as body language. This type of communication is very important in the doctor-patient relationship due to factors such as the limited visiting time, and linguistic and discourse differences.

**3. **Truth-Telling versus Pain Relief: One of the oldest ethical challenges is the pain and suffering that can be caused by telling the truth. On the other hand, we can bring comfort and relief to patients by lying to them. Physicians can employ various methods at their discretion, but it seems that health care systems are more inclined toward telling the truth, and doctors must try to maintain a balance between the two.

**4. **Emotional Quotient (EQ): Unlike intelligence quotient that does not improve after the second decade of life, emotional quotient can continue to improve till the end. Emotional quotient refers to the ability to control emotions, sentiments and unwanted desires. People with high intelligence quotient dealing with people with lower intelligence quotient are susceptible to reckless, impulsive behavior and may gradually lose their EQ (10). In order to improve the doctor-patient relationship, health providers must be instructed in techniques to promote their emotional quotient. 

**C) The Sociological Essence of the Doctor-Patient Relationship**

Unlike the psychological approach, the sociological approach to the doctor-patient relationship examines the essence of this (individualistic) relationship in a social context. In other words, the sociological approach regards the doctor-patient relationship beyond a merely mutual connection and therefore external elements are considered particularly important.

In order to investigate this relationship from the sociological perspective, communicative actions serve as a valid basis. They have been included among the most important sociological criteria in the last few decades as a set of social actions oriented towards reaching entente. The target of communication action theory is to subvert a single prophetic and patriarchal individualism in human interactions. Jürgen Habermas has developed this notion in his famous book *The Theory of Communicative Action*, and his ideas are quite often presented in ethical manuscripts and medical ethics books. In this book Habermas distinguishes and characterizes his theory by drawing a distinction between instrumental action and communicative action. 

***Instrumental Action***

Jürgen Habermas states, “We call an action oriented to success instrumental when we consider it in the light of following the rules of rational choice and assess the efficiency of influencing the decisions of a rational opponent. By contrast, I shall speak of communicative action whenever the actions of the agents involved are coordinated not through egocentric calculations of success but through acts aiming at reaching an understanding. In communicative action the participants are not primarily oriented to their own individual successes; they pursue their individual goals under the condition that they can harmonize their plans of action on the basis of common situation definitions. In this respect, negotiating the definitions of the situation is an essential element of the interpretive accomplishments required for communicative action” (11). 

Reducing an individual to only one of the functions of his or her integrity is called instrumentalism. The function of a ticket seller in a bus station is just like that of a machine and therefore his human dimension could easily be overlooked. In the doctor-patient relationship both sides (especially the doctor) are susceptible to perceive others as mere instruments. The power that is practiced over patients by "medical gazing" makes them abject by reducing them to bodies that are examined simply to locate illness. Three fundamental concepts in sociology and philosophy have been purposed to deal with instrumentalism: 

*1) Teleological view of others by emphasizing the task:*

In his works on the Golden Rule, Kant argues that instrumental action is inconsistent with socialization and human dignity, and proposes to regard others as an acme, not an instrument. The universal version of this rule is that you should like for others whatever you like for yourself and vice versa. One concrete technique for applying this rule is that human beings constantly put themselves in other people’s positions and see the world from their perspectives.

*2) The distinction between mysterious looks and issue makers:*

Martin Buber and Gabriel Marcel emphasize the difference between the I-Thou and I-It relationship. In the former, a human is a mystery that unfolds and in the latter, an issue that resolves (12).

*3) Maximum responsibility toward others:*

Emmanuel Levinas states, "We are responsible for each other, and me more so…" (13). This approach considers responsibility toward others as an unconditional matter, but does not require others to be equally responsible in return. 

***Communicative Action***

Communicative action is allegedly an action focused on entente. Whoever wants to be successful in reaching entente should be prepared to bring up claims. Habermas states that the communication between a speaker and a listener is constituted by the existence of three universally valid claims: the claims for truth, rightness and truthfulness (11). The terms of these claims in the doctor-patient relationship accurately reveal the sociological essence of this relationship. Doctors should speak understandably and beware of ambiguity and opacity in their speech. On the other hand, they should make true statements and propositions, scientific and other. They should be honest and have faith in what they say, and ultimately they can use their discretion to determine the content of their relationship with patients. 

***Analysis***

To clarify the concept of relationship and connectedness, we used a hybrid concept analysis including: identifying essential attributes, critiquing the existing definitions, examining boundaries and identifying antecedents (14). On the basis of the comparative concept analysis, the doctor-patient relationship is an interdisciplinary notion and a mono-disciplinary approach will reduce this relationship to communicative skills. 

## Discussion and Conclusion

The doctor-patient relationship has greater impact on the health system than it may seem at first. In this paper, three novel dimensions of the doctor-patient relationship were deeply explored. The philosophical approach emphasizes the importance of promoting moral sensitivity. Communicating with others entails considerations rooted in the human soul that provoke great philosophical concerns. The psychological approach emphasizes learning about behavioral patterns, enhancing the intelligence quotient, and creating a balance between truth-telling and pain relief. Finally, the sociological approach demonstrates that the doctor-patient relationship is part of a macro social relationship in a community and discovers various aspects beyond the two-person relationship. 

The re-examination of the doctor-patient relationship in this paper can have several important implications. Attention to the philosophical, sociological and psychological dimensions provides a basis to evaluate the doctor-patient relationship both quantitatively and qualitatively. Two well-known examples of such qualitative and quantitative evaluations may be seen in the development of native questionnaires and conversion of random considerations to systemic approaches. 

As a final word, a re-examination of the doctor-patient relationship requires an interdisciplinary approach that should take into account the legal as well as juridical essence in addition to the three approaches discussed in this paper.
